# The application of podocyte antigen PLA2R and anti-PLA2R antibody in the diagnosis and treatment of membranous nephropathy

**DOI:** 10.1080/0886022X.2023.2264939

**Published:** 2023-10-09

**Authors:** Yang Chen, Ying Xu, Siyu Chen, Yedong Yu, Xueling Zhu, Jianghua Chen

**Affiliations:** a Kidney Disease Center, the First Affiliated Hospital, College of Medicine, Zhejiang University, Hangzhou, Zhejiang, China; b Key Laboratory of Kidney Disease Prevention and Control Technology, Hangzhou, Zhejiang, China; c National Key Clinical Department of Kidney Diseases, Hangzhou, Zhejiang, China; d Institute of Nephrology, Zhejiang University, Hangzhou, Zhejiang, China; e Zhejiang Clinical Research Center of Kidney and Urinary System Disease, Hangzhou, Zhejiang China; f Zhejiang Provincial People’s Hospital, Hangzhou, Zhejiang, China; g The Department of Infectious Diseases, State Key Laboratory for Diagnosis and Treatment of Infectious Diseases, National Clinical Research Center for Infectious Diseases, Collaborative Innovation Center for Diagnosis and Treatment of Infectious Diseases, The First Affiliated Hospital, School of Medicine, Zhejiang University, Hangzhou, China

**Keywords:** Membranous nephropathy, PLA2R, anti-PLA2R, diagnosis, prognosis

## Abstract

**Background:**

The application of podocyte antigen M-type phospholipase A2 receptor (PLA2R, GAg) and serum anti-PLA2R antibody (SAb) in predicting the prognosis of membrane nephropathy (MN) was controversial.

**Method:**

328 biopsy-proven MN patients were divided into three phenotypes, 182 MN patients with GAg+/SAb+, 118 MN patients with GAg+/SAb-, and 28 MN patients with GAg-/SAb-. The baseline clinicopathological characteristics, therapy response, and prognosis were compared among the three groups. Cox regression analysis was performed to assess predictors of remission. Anti-PLA2R antibody was analyzed by receiver operating characteristic curve to find the optimal titer for MN diagnosis.

**Result:**

Lower eGFR (*p* = 0.009), higher UPCR (*p* < 0.001), and lower serum albumin (*p* < 0.001) were observed in GAg+/SAb+ MN patients, compared to GAg+/SAb- MN patients. More GAg+/SAb+ MN patients received cyclophosphamide (CTX) combined with glucocorticoids and calcineurin inhibitors (CNI) based therapy than the other two groups (*p* = 0.015 and *p* = 0.023, respectively). No significant difference was observed among the three groups in terms of complete remission, relapse, and developing ESRD. SAb+ status was an independent predictor for no remission (hazard ratio 1.378, 95% confidence interval 1.023 to 1.855; *p* = 0.035). The optimal cutoff value for anti-PLA2R antibody to predict MN was 2.055 RU/mL (sensibility 0.802, specificity 0.970).

**Conclusion:**

GAg+/SAb+ MN patients were related to more severe clinical manifestations and more requisition of immunosuppressive treatment. Positive anti-PLA2R antibody was an independent predictor for no remission. An anti-PLA2R antibody above 2.055 RU/mL can be a suggestive indicator of MN diagnosis in patients with proteinuria.

## Introduction

Membranous nephropathy (MN) is a common etiology of nephrotic syndrome in adults, as well as a major contributor to end-stage renal disease (ESRD). Due to environmental pollution, the incidence of MN has been increasing year by year [1]. Patients with MN account for 23.4% of primary glomerular diseases in China, ranking second among primary glomerular diseases [1]. Though spontaneous remissions are common in MN, 35% of untreated patients with nephrotic syndrome will develop ESRD at 10 years [2]. Precisely predicting the prognosis of MN patients combined with effective treatment and management strategies are crucial [3].

The discovery of the M-type phospholipase A2 receptor (PLA2R) in 2009 greatly motivated basic and clinical research in MN, which has been widely applied as a biomarker for the diagnosis and prognosis prediction of MN over the past 10 years [4,5]. PLA2R-associated MN is defined as MN patients with either positive staining for PLA2R antigen in the glomeruli (GAg) or positive serum anti-PLA2R antibodies (SAb) [6]. In the diagnosis test, tissue immunostaining for PLA2R, either by immunofluorescence (IF) or immunohistochemistry (IHC), is more sensitive (sensitivity 69 to 84%) for diagnosing primary MN than detecting circulating anti-PLA2R antibody (sensitivity 57 to 82%) [1,7–9]. Assessing the accuracy and optimal cutoff levels of SAb for the diagnosis of MN is a research opportunity. Besides, the application of GAg and SAb in predicting remission of MN is controversial. One retrospective research followed 52 primary MN patients and found that patients who did not achieve remission experienced a higher positive rate of GAg and SAb at the first biopsy than those who achieved remission [10]. Further analysis found positive SAb and persistent GAg expression were correlated with no remission [10]. Nevertheless, another research with a sample size of 51 indicated the cumulative remission rate was comparable between MN patients with or without GAg expression [9]. Besides, Yun Jung Oh et al. showed there was no significant correlation between the levels or presence/absence of SAb and remission[11].

This study retrospectively analyzed the baseline clinicopathological characteristics, therapy response, and prognosis of primary MN patients, grouped as GAg+/SAb+, GAg+/SAb- and GAg-/SAb-, and aimed to explore the relationship between GAg/SAb and prognosis of MN and the optimal cutoff level of SAb for the diagnosis of MN.

## Methods

### Study population

**Figure 1.**
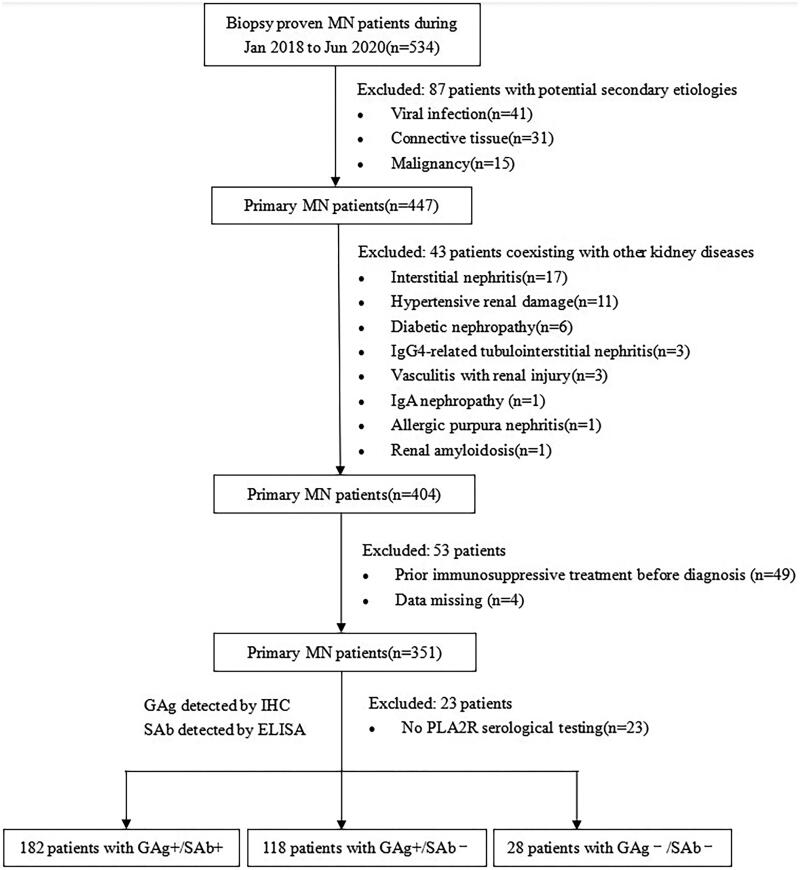
Flowchart of patient inclusion.

Between January 2018 and June 2020, 534 patients were diagnosed pathologically with MN in Kidney Disease Center, the First Affiliated Hospital, College of Medicine, Zhejiang University. Among those, 87 patients had potential secondary etiologies of MN, including viral infection (*n* = 41), autoimmune disease (*n* = 31) and malignancy (*n* = 15). Forty-three patients coexisted with other kidney diseases, including interstitial nephritis (*n* = 17), hypertensive renal damage (*n* = 11), diabetic nephropathy (*n* = 6), vasculitis with renal injury (*n* = 3), IgG4-related tubulointerstitial nephritis (*n* = 3), IgA nephropathy (*n* = 1), Allergic purpura nephritis (*n* = 1) and renal amyloidosis (*n* = 1). Forty-nine patients received prior immunosuppressive treatment before diagnosis. Four patients were excluded for missing data and 23 patients without PLA2R serological test were excluded. Therefore, 328 individuals were included (Figure 1).

The included patients had SAb tested by enzyme-linked immunosorbent assay (ELISA) and GAg detected by IHC on paraffin tissue. Among the included patients, 182 patients were verified with glomerular PLA2R expression and positive serum anti-PLA2R antibody (GAg+/SAb+), 118 patients with glomerular PLA2R expression but negative serum anti-PLA2R antibody (GAg+/SAb-), 28 patients with negative glomerular PLA2R expression and negative serum anti-PLA2R antibody (GAg-/SAb-). Follow-up time ended on 30 July 2023, with a median follow-up of 37 months.

Besides, we included biopsy-proven non-MN patients between January 2019 to June 2020 as a control cohort for the diagnostic test. The inclusion criteria were: 1) the pathological diagnosis was IgA nephropathy (IgAN), minimal change disease (MCD), focal segmental glomerulosclerosis (FSGS) or diabetic nephropathy (DN); 2) underwent PLA2R serological test at the time of renal biopsy. The exclusion criterion was coexisting with MN.

### Clinicopathological data

Demographic characteristics, clinical manifestations, laboratory parameters, and renal biopsy characterization of each participant were recorded. The number of patients with missing data for each variable is shown in Table S1. Estimated glomerular filtration rate (eGFR) was calculated according to the CKD-EPI formula [12]. Urinary protein-to-creatinine ratios (UPCR) were used to assess urinary protein excretion in place of 24-h urine protein. Basic renal lesions were evaluated [13]. The number of patients with focal segmental glomerulosclerosis (FSGS), crescent lesions, or moderate/severe mesangial hyperplasia was recorded. Interstitial fibrosis was described by the percent of the fibrotic area versus gross interstitial. Stages of MN were defined Using Ehrenreich and Churg’s criteria with transmission electron microscopy.

### SAb measurement, GAg detection, and immunostaining

**Figure 2.**
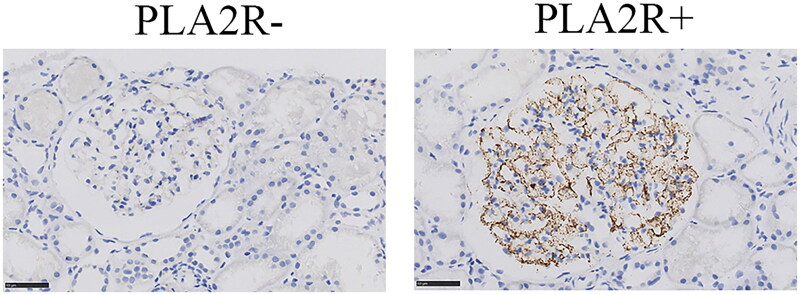
Immunohistochemical staining on paraffin tissue for PLA2R in MN patients. PLA2R expressing in a fine, granular pattern along the glomerular capillary at high magnification was considered positive. Bars= 50µm.

Serum anti-PLA2R antibody was measured at the time of renal biopsy by antigen-specific ELISA using PLA2R as the antigen (ELISA kits were bought from EUROIMMUN). Serum anti-PLA2R antibody higher than 20 RU/mL is defined as positive according to the manufacturer. IHC on paraffin tissue by antibodies targeting PLA2R (1:30000, ab211490, Abcam) was performed to assess glomerular PLA2R expression. PLA2R expressing in a fine, granular pattern along the glomerular capillary at high magnification was considered positive (Figure 2).

Immunoglobulin A (IgA), Immunoglobulin G (IgG), immunoglobulin M (IgM), complement 3 (C3), complement 4 (C4), and complement (C1q) were examined by IF on frozen sections. Primary antibodies were bought from Gene Tech and the dilution ratio was 1:30. IgG subclasses were examined using IHC on paraffin sections. Primary antibodies were bought from Gene Tech and dilution ratio was 1:50. The criteria for immunostaining scoring were as follows: 0, negative (no staining even at high magnification); 1, weak (visible only at high magnification); 2, moderate (visible at low magnification); 3, strong (strikingly positive even at low-power magnification).

### Treatment options and outcome definitions

Treatment strategies and prognosis were recorded. Treatment regimens were selected based on risk evaluation according to KIDGO guidelines [14]. Immunosuppressive treatment was administered either after a 6-month observation period with supportive care in patients with partial remission or no response, or earlier, in patients at high risk for progressive kidney injury. Immunosuppressive regimens included cyclophosphamide (CTX) with glucocorticoids, calcineurin inhibitors (CNI) based therapy, CD20-targeted therapy, mycophenolate mofetil combined with glucocorticoids and others like Chinese herbal medicine like Tripterygium. Complete remission was defined as urinary protein excretion < 0.3 g/g with normal serum albumin, and partial remission was defined as a 50% or greater reduction in urinary protein with urinary protein excretion between 0.3 and 3.5 g/g [15]. Relapse was defined as a return of proteinuria to > = 3.5 g/g during an initial complete or partial remission [14]. Developing end-stage renal disease was defined as an eGFR < 15 mL/min/1.73m^2^.

### Statistical analysis

The software SPSS (version 26.0) was used for statistical analysis. Numerical data with skewed distribution were expressed as the median and interquartile range (IQR), and assessed by non-parametric test among the three groups. Kruskal-Wallis test was used for pairwise comparison. Categorical data were summarized as percentages and compared by χ^2^ tests followed by Bonferroni correction for pairwise comparison. *P* value or adjusted *p*-value less than 0.05 was considered statistically significant. Kaplan-Meier analysis based on IHC was conducted to analyze cumulative remission rates using the log-rank method. Cox proportional hazards models were used to determine the independent prognostic factors of remission. Variables with *p* < 0.1 in univariate analyses were enrolled in multivariate analyses, provided that there was no presence of collinearity. Furthermore, the multivariable analyses incorporated GAg, proteinuria, eGFR, and interstitial fibrosis, as they have been established as significant factors influencing MN outcome according to the published evidence[16,17]. To determine the precise titer of serum anti-PLA2R antibody for predicting the diagnosis of MN, we performed a receiver operating characteristic curve and found the cutoff value according to the maximum of the Youden index (specificity + sensitivity-1).

## Results

A total of 328 primary MN patients were enrolled in the study. The cohort included 202 (61.59%) men with the median age of 53 years at diagnosis. Most patients (94.51%) had preserved renal function (eGFR ≥ 60 mL/min/1.73m^2^) with a median eGFR of 95.35 mL/min/1.73m^2^. 82 (25.54%) patients had nephrotic syndrome with a median proteinuria of 2.44 g/g, and a median albuminemia of 26.30 g/L.

### Baseline clinicopathological characteristic

The baseline clinicopathological characteristic of patients at enrollment are shown in Table 1. GAg+/SAb+ MN patients tended to have higher systolic blood pressure (SBP) than GAg+/SAb- MN patients and GAg-/SAb- MN patients (*p* = 0.045 and *p* = 0.017, respectively), higher diastolic blood pressure than GAg-/SAb-MN patients (*p* = 0.016). Lower eGFR (*p* = 0.009), higher UPCR (*p* < 0.001), and lower serum albumin (*p* < 0.001) were observed in GAg+/SAb+ MN patients, compared to GAg+/SAb- MN patients. The proportion of nephrotic-range proteinuria and nephrotic syndrome significantly differed among the three groups (*p* = 0.021 and *p* = 0.017, respectively). GAg+/SAb+ MN patients exhibited higher serum cholesterol concentrations than GAg+/SAb- MN patients and GAg-/SAb- MN patients (*p* < 0.001 and *p* = 0.008, respectively). Besides, more GAg+/SAb+ MN patients and more GAg+/SAb- MN patients presented microscopic hematuria than GAg-/SAb- MN patients (*p* = 0.007). After Bonferroni correction, there were no significant differences in demographic data and serum creatinine among the three groups.

**Table 1.** Baseline clinicopathological characteristics, treatment and prognosis in GAg+/SAb+ MN patients, GAg+/SAb- MN patients and GAg-/SAb- MN patients.

	MN patients (*n* = 328)	MN patients with GAg+/SAb+ (*n* = 182)	MN patients with GAg+/SAb- (*n* = 118)	MN patients with GAg-/SAb- (*n* = 28)	P value
Clinicopathological parameters					
Male (n)	202(61.59%)	109(59.89%)	79(66.95%)	14(50.00%)	0.197
Age (year)	53.00(42.00–62.00)	54.00(45.00–62.00)	51.00(36.25–59.00)	57.00(39.25–63.75)	0.071
SBP (mmHg)	130.00 (120.00–142.00)	132.00 (121.50–145.50)	126.00(116.75–139.25)*	120.50(112.50–137.25)^#^	0.004
DBP (mmHg)	80.00(72.00–89.00)	81.00(73.00–90.00)	80.00(72.00–85.50)	74.50(71.00–79.75)^#^	0.011
Serum creatinine (μmol/L)	73.00(59.00–85.00)	74.50(62.00–86.25)	72.00(57.00–82.25)	68.00(51.00–79.00)	0.036
eGFR (mL/min/1.73m^2^)	95.35(86.52–108.62)	94.17(82.19–103.65)	98.35(89.80–113.25)*	96.73(87.95–115.02)	0.009
Kidney function stage (n)					0.138
eGFR < 60 mL/min/1.73m^2^	18(5.49%)	14(7.69%)	3(2.54%)	1(3.57%)	
eGFR ≥ 60 mL/min/1.73m^2^	310(94.51%)	168(92.31%)	115(97.46%)	27(96.43%)	
UPCR (g/g)	2.44(1.42–3.67)	2.76(1.78–4.21)	1.94(0.96-3.25)*	2.25(1.03–4.12)	<0.001
Nephrotic-range proteinuria (n)	93(28.97%)	63(34.81%)	22(19.64%)*	8(28.57%)^#※^	0.021
Nephrotic syndrome	82(25.54%)	56(30.94%)	18(16.07%)*	8(28.57%)^#※^	0.017
Serum albumin (g/L)	26.30(22.03–31.35)	24.50(21.10–28.53)	29.50(25.53–34.65)*	28.10(20.33–28.53)	<0.001
Cholesterol (mmol/L)	6.18(5.03-7.54)	6.51(5.55-8.11)	5.70(4.65–6.89)*	5.20(4.47–6.99)^#^	<0.001
Hematuria (n)	126(38.41)	76(41.76)	47(39.83)	3(10.71)^#※^	0.007
The number of Glomeruli	27.50(20.00–38.75)	27.00(19.75–38.00)	30.00(21.00–40.00)	26.00(15.00–34.75)	0.212
FSGS (n)	13(3.96%)	9(4.95%)	4(3.39%)	0(0.00%)	0.629
Crescent (n)	9(2.74%)	4(2.20%)	3(2.54%)	2(7.14%)	0.305
Moderate/severe mesangial hyperplasia (n)	6(1.83%)	2(1.10%)	2(1.69%)	2(7.14%)	0.106
Interstitial fibrosis (%)	5.00(5.00–5.00)	5.00(5.00–5.00)	5.00(5.00–5.00)	5.00(5.00–5.00)	0.124
Immunofluorescence^@^(n)					
IgA	23(7.06%)	11(6.04%)	10(8.62%)	2(7.14%)	0.698
IgG	249(75.9%)	143(78.57%)	91(77.12%)	15(53.57%)^#※^	0.015
IgM	27(8.28%)	11(6.04%)	15(12.93%)	1(3.57%)	0.070
C3	48(14.72%)	31(17.03%)	15(12.93%)	2(7.14%)	0.309
C4	1(0.31%)	0(0%)	1(0.86%)	0(0%)	0.442
C1q	2(0.61%)	1(0.55%)	0(0%)	1(3.57%)	0.165
Immunohistochemistry^@^(n)					
IgG1	134(41.36%)	78(43.09%)	45(39.13%)	11(39.29%)	0.775
IgG2	0(0%)	0(0%)	0(0%)	0(0%)	NA
IgG3	3(0.93%)	3(1.66%)	0(0%)	0(0%)	0.455
IgG4	158(48.76%)	95(52.49%)	59(51.30%)	4(14.29%)^#※^	0.001
Electron-dense deposits sites(n)					
Subepithelial areas	262(81.62%)	154(86.03%)	88(76.52%)	20(74.07%)	0.069
Intramembranous areas	200(62.31%)	91(50.84%)	93(80.87%)*	16(59.26%)^#※^	<0.001
Mesangial areas	153(47.66%)	73(40.78%)	69(60.00%)*	11(40.74%)^#※^	0.004
Subendothelial areas	3(0.93%)	1(0.56%)	0(0%)	2(7.41%)^#※^	0.019
The Ehrenreich-Churg stage(n)					0.054
Stage I-II	283(86.54%)	163(90.01%)	99(83.90%)	21(75.00%)	
Stage III-IV	44(13.46%)	18(9.94%)	19(16.10%)	7(25.00%)	
Treatment and prognosis					
Patients lost to follow-up (n)	39(9.90%)	18(9.89%)	20(16.95%)	1(3.57%)	0.076
Treatment (n)					
Supportive treatment	55(16.87%)	21(11.67%)	25(21.19%)*	9(32.14%)^#※^	0.008
CTX combined with GCs	33(10.12%)	26(14.44%)	6(5.08%)*	1(3.57%)^#※^	0.015
CNI based therapy	118(36.20%)	77(42.78%)	33(27.97%)*	8(28.57%)^#※^	0.023
CD20-targeted therapy	20(6.13%)	12(6.67%)	8(6.78%)	0(0%)	0.442
MMF combined with GCs	3(0.92%)	1(0.56%)	1(0.85%)	1(3.57%)	0.283
Others^&^	58(17.79%)	25(13.89%)	25(21.19%)	8(28.57%)	0.077
Prognosis (n)					
No remission	69(21.04%)	41(22.53%)	21(17.80%)	7(25.00%)	0.534
Partial remission	93(28.35%)	61(33.52%)	24(20.34%)*	8(28.57%)^#※^	0.047
Complete remission	127(38.72%)	62(34.07%)	53(44.92%)	12(42.86%)	0.152
Time to partial remission (m)	10.50(5.25–22.50)	11.00(6.00–24.00)	12.00(6.00–12.00)	8.00(2.75–22.50)	0.734
Time to complete remission (m)	12.00(8.00–24.00)	14.00(10.00–29.75)	10.00(6.75-–20.25)	11.00(5.25–12.75)	0.037
Relapse(n)	32(9.76%)	19(10.44%)	12(10.17%)	1(3.57%)	0.661
ESRD(n)	7(2.15%)	6(3.33%)	1(0.85%)	0(0%)	0.392
Follow-up time (m)	37.00(22.00-48.00)	37.00(23.00-47.00)	38.00(25.75–51.00)	36.00(22.00–53.00)	0.422

GAg: glomerular PLA2R expression; SAb: serum anti-PLA2R antibody; SBP: systolic blood pressure; DBP: diastolic blood pressure; eGFR: estimated glomerular filtration rate; UPCR: urinary protein creatinine ratio; FSGS: focal segmental glomerulosclerosis; GCs: glucocorticoids; *significant difference between GAg+/SAb+ MN patients and GAg+/SAb- MN patients; # significant difference between GAg+/SAb+ MN patients and GAg-/SAb- MN patients; ※significant difference between GAg+/SAb- MN patients and GAg-/SAb- MN patients; @ Moderate positive staining, which means visible at low magnification, is defined as immunoglobulin or complement deposition. & others included Chinese herbal medicine like tripterygium.

The number of glomeruli, the number of patients with FSGS, crescent, moderate/severe mesangial hyperplasia, and the percentage of interstitial fibrosis (IF) was comparable among the three groups (Table 1). The deposition of IgG and IgG4 was less commonly seen in GAg-/SAb- MN patients than in GAg+/SAb+ MN patients and GAg+/SAb- MN patients (*p* = 0.015 and *p* = 0.001, respectively). However, the proportion of intramembranous and mesangial deposits on electron microscopy differed significantly between the three groups (*p* < 0.001 and *p* = 0.004, respectively). Subendothelial electron-dense deposits were more common in GAg-/SAb- MN patients than GAg+/SAb+ MN patients and GAg+/SAb- MN patients (*p* = 0.019). There was no significant difference in the number of patients at stage I-II among the three groups.

### Treatment and prognosis

There was no significant difference in the number of patients lost to follow-up among the three groups (Table 1). More GAg+/SAb + MN patients received cyclophosphamide (CTX) combined with glucocorticoids and calcineurin inhibitors (CNI) based therapy than GAg+/SAb- MN patients and GAg-/SAb- MN patients (*p* = 0.015 and *p* = 0.023, respectively). Less GAg+/SAb + MN patients received supportive treatment than GAg+/SAb- MN patients and GAg-/SAb- MN patients (*p* = 0.008). More GAg+/SAb + MN patients achieved partial remission than GAg+/SAb- MN patients and GAg-/SAb- MN patients (*p* = 0.047). No significant difference was observed among the three groups in terms of complete remission, relapse, and developing ESRD.

### Predictors for remission

We performed Kaplan-Meier analysis based on GAg to compare cumulative remission rates. There is no significant difference in the cumulative remission rates or complete remission rates between patients with or without GAg (Figure S1). The results of the univariable cox regression analysis indicated that SAb+ status was a risk factor of lack of remission in MN patients (hazard ratio 1.272, 95% confidence interval 0.972 to 1.664; *p* = 0.079) (Table 2). After adjusting for GAg+ status, proteinuria, eGFR, and interstitial fibrosis, SAb+ remained an independent risk factor for lack of remission (hazard ratio 1.378, 95% confidence interval 1.023 to 1.855; *p* = 0.035).

**Table 2.** Predictors for lack of remission in Univariable and multivariable Cox regression analysis.

Predictor	Univariable Analysis		Multivariable Analysis*	
	HR (95% CI)	p	HR (95% CI)	*p*
GAg+ vs GAg-	1.121 (0.707–1.778)	0.627	0.991 (0.604–1.627)	0.973
SAb+ vs SAb-	1.272 (0.972–1.664)	0.079	1.378 (1.023–1.855)	0.035
Age, per 1 year older	1.006 (0.997–1.016)	0.191	–	–
Male sex	0.843 (0.645–1.103)	0.214	–	–
UPCR, per 1 g/g greater	0.973 (0.905–1.045)	0.449	0.965 (0.894–1.042)	0.364
eGFR, per 1 mL/min/1.73m^2^ greater	1.000 (1.000–1.000)	0.442	1.000 (1.000–1.000)	0.350
Interstitial fibrosis, per 1% greater	0.987 (0.959–1.015)	0.360	0.990 (0.960–1.021)	0.537

GAg: glomerular PLA2R expression; SAb: serum anti-PLA2R antibody; UPCR: urinary protein creatinine ratio; eGFR: estimated glomerular filtration rate; * Adjusted for glomerular PLA2R expression, proteinuria, eGFR, and interstitial fibrosis.

### The optimal cutoff value of anti-PLA2R antibody to diagnose MN

**Figure 3.**
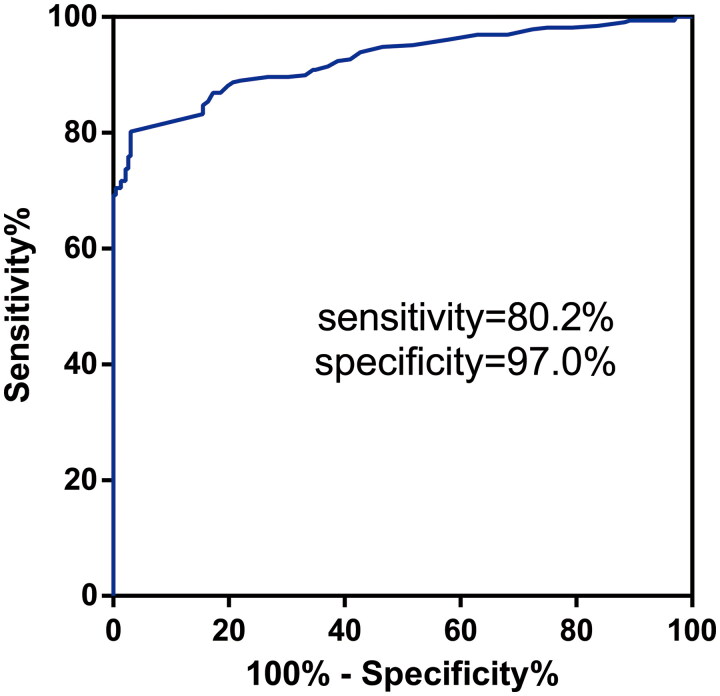
ROC analysis of anti-PLA2R antibody.

To explore the optimal cutoff value of anti-PLA2R antibody to diagnose MN, we included 328 biopsy-proven MN patients and 232 biopsy-proven non-MN patients (96 IgAN patients, 80 MCD patients, 22 FSGS patients, 34 DN patients). We applied the ROC curve to determine the optimal titer of SAb to predict MN (Figure 3). The area under ROC curve was 0.926 (95% confidence interval 0.904–0.947, *p* < 0.0001, sensitivity= 0.802, specificity= 0.970). Based on the maximum value of the Youden index, the cutoff value was 2.055 RU/mL for anti-PLA2R antibody.

## Discussion

In the study, we found that GAg+/SAb+ is a characteristic of more severe conditions and more requisition of immunosuppressive treatment in MN. However, SAb+ rather than GAg+ was identified as an independent risk factor for lack of remission. To help the noninvasive diagnosis of MN, the optimal cutoff value of anti-PLA2R antibody, 2.055 RU/mL, was determined.

Primary membrane nephropathy is considered an autoimmune disease with exclusive kidney involvement, and anti-PLA2R antibody is the dominating pathogenic autoantibody. In our study, among GAg+ MN patients, 60.7% tested positive for anti-PLA2R antibody, whereas 39.3% tested negative despite positive staining of PLA2R in the tissue. The proportion was consistent with previous studies [8,9]. The possible mechanism may be that podocyte antigen in the kidney binds with an antibody with a high affinity, so only when the interaction reaches saturation, the anti-PLA2R antibody can be detected in the peripheral [5].

In the cohort, the proportions of nephrotic-range proteinuria and nephrotic syndrome seemed to be lower than that reported previously, possibly due to universal physical examination screening and early detection of MN. On the other hand, the proportion of patients with hematuria was consistent with previous reports that microscopic hematuria occurs in up to 50% of MN patients [18]. For pathological characteristics, Subepithelial IgG and C3 deposition is a typical pathological feature of primary MN, and anti-PLA2R antibodies are predominantly of the IgG4 isotype [4]. However, IgG and IgG4 deposition was less common in GAg-/SAb- MN patients, indicating the possibility of secondary MN and new podocyte target antigen. Except PLA2R, new target antigens like thrombospondin type-1 domain-containing 7 A (THSD7A), neural epidermal growth factor-like 1 (NELL1), exostosin-1/exostosin-2 (EXT1/EXT2), semaphorin 3B (Sema3B) and protocadherin 7 (PCDH7) were identified successively [19–24]. Atypical pathological characteristics including subendothelial deposition were more common in GAg-/SAb- MN patients, suggesting the possibility of undiscovered secondary MN. Previous investigations correlated the histological chronicity indices like FSGS, IF, and vascular hyalinosis (VH) with response to immunosuppressive therapy [17]. Nevertheless, this conclusion was not validated in our study. Most patients in our cohort were at EM stage I-II with little interstitial fibrosis, suggesting they were in the immunologic active state rather than the chronic phase at the diagnosis [25]. Hence, the results from our cohort would have a limitation when assessing the relationship between chronicity score and outcomes.

Discrepancies regarding the relationship between SAb and remission exist in previous studies. Early studies emphasized high titer of anti-PLA2R in serum at diagnosis was related to an increased risk of progressive loss of renal function, the rapid transformation from non-nephrotic-range proteinuria to nephrotic-range proteinuria, and a decreased spontaneous remission rate [26–31]. While some studies found no correlation between the baseline titer of SAb and remission[11, 32]. Our analysis showed the presence of SAb was an independent risk factor for no remission. Possible explanation for the heterogeneity may be the selection of participants and variable form of SAb (as a continuous variable or a categorical variable). In patients with seropositive PLA2R-associated MN, the titer of SAb was not relevant to remission, however, SAb+ status was an independent predictor for lack of remission in the entire primary MN population. Consistently, in 182 seropositive PLA2R-associated MN in our cohort, the titer of SAb was not an independent predictor. Similarly, the relationship between GAg and remission was controversial in different populations. In patients with seropositive PLA2R-associated MN, GAg- is independently associated with poor response to treatment [16]. However, Qin et al. found persistent GAg deposits were correlated with a poor response to treatment in primary MN [10]. Our study showed GAg status did not significantly influence remission, consistent with Li’s findings [9].

We found that the sensitivity of serum anti-PLA2R antibody in the diagnostic test for diagnosing MN was only 55.5% (182/328) in our cohort. We expected to improve the performance of serum anti-PLA2R antibodies by exploring the optimal cutoff value. Though the threshold of anti-PLA2R antibody used in most centers is 20 RU/mL, some experts suggest redefining serological positivity as anti-PLA2R antibody detected by ELISA exceeding 2-3 RU/mL (much lower than 20 RU/mL) [33–36], to increase sensitivity without a reduction of specificity. In our study, sensitivity and specificity were 0.555 and 1.0 respectively when the threshold was defined as 20 RU/mL, however, sensitivity reached 0.802 with specificity remaining at 0.970 if we adjusted the threshold to 2.055 RU/mL. Nevertheless, an earlier study using the same assay method showed better diagnostic capability when defining the cutoff value of anti-PLA2R antibody as 20 RU/mL rather than 2 RU/mL [37]. The contrasting results in this regard may be due to different time points when the anti-PLA2R antibody was examined. As immunosuppressants may change the titer of anti-PLA2R antibody, we measured the titer of anti-PLA2R antibody at the time of renal biopsy and excluded patients with prior immunosuppressive therapy. In addition, Bobart et al. proposed the strategy to make a noninvasive diagnosis of MN when patients tested positive for serum anti-PLA2R antibody in the setting of preserved renal function and no potential secondary causes [36]. The diagnostic algorithm was also applicable to our cohort since the kidney biopsy did not provide significant information that altered management in patients fulfilling the above criteria. This strategy may improve early noninvasive diagnosis for MN, especially for those nephrotic syndrome patients with a contradiction for kidney biopsy.

There are some limitations in this study. Firstly, the titer of anti-PLA2R during the follow-up was not available in this study, and a dynamic process of immunologic activity could not be observed. Secondly, no patients presented GAg-/SAb+ in our cohort [16], so we were unable to summarize the characteristics of the distinct subtype.

In conclusion, MN patients with positive glomerular PLA2R expression and positive anti-PLA2R antibody were related to more severe clinical manifestations and more requisition of immunosuppressive treatment. Besides, positive anti-PLA2R antibody, rather than positive glomerular PLA2R expression, was identified to have a negative effect on remission significantly. An anti-PLA2R antibody above 2.055 RU/mL can be a reference for MN diagnosis in patients with proteinuria.

### Ethics approval and consent to participate

This study was performed in line with the principles of the Declaration of Helsinki. Approval was granted by the Ethics Committee of the First Affiliated Hospital, Zhejiang University School of Medicine, approval number 2022-1025.The study was granted an exemption from requiring written informed consent by the Ethic Committee of the First Affiliated Hospital, Zhejiang University School of Medicine.

## Supplementary Material

Supplemental Material

## Data Availability

The datasets generated and analyzed during the current study are available from the corresponding author on reasonable request.
