# Supplementary material for: The application of podocyte antigen PLA2R and anti-PLA2R antibody in the diagnosis and treatment of membranous nephropathy
Source: Ren Fail. 2023 Oct 9;45(2):2264939. doi: 10.1080/0886022X.2023.2264939 (PMC10566392; doi:10.1080/0886022X.2023.2264939)
Supplement: Supplemental Material [file IRNF_A_2264939_SM4021.pdf]

Table S1. The number of patients with missing data for each variable

Number of patients(n)	MN patients (n=328)	MN patients with GAg+/SAb+ (n=182)	MN patients with GAg+/SAb- (n=118)	MN patients with GAg-/SAb- (n=28)
Clinicopathological parameters				
Male	0	0	0	0
Age	0	0	0	0
SBP	1	1	0	0
DBP	2	1	1	0
Serum creatinine	0	0	0	0
eGFR	0	0	0	0
UPCR	7	1	6	0
Serum albumin	0	0	0	0
Cholesterol	0	0	0	0
Hematuria	1	1	0	0
The number of Glomeruli	0	0	0	0
FSGS	0	0	0	0
Crescent	0	0	0	0
Moderate/severe mesangial hyperplasia	0	0	0	0
Interstitial fibrosis	0	0	0	0

Information Classification: General

Table S1. (continued).

	MN patients (n=328)	MN patients with GAg+/SAb+ (n=182)	MN patients with GAg+/SAb- (n=118)	MN patients with GAg-/SAb- (n=28)
Immunofluorescence				
IgA	2	0	2	0
IgG	0	0	0	0
IgM	2	0	2	0
C3	2	0	2	0
C4	2	0	2	0
C1q	2	0	2	0
Immunohistochemistry				
IgG1	4	1	3	0
IgG2	4	1	3	0
IgG3	4	1	3	0
IgG4	4	1	3	0
Electron-dense deposits sites	7	3	3	1
The Ehrenreich-Churg stage	1	1	0	0
Treatment and prognosis				
Patients lost to follow-up	39	18	20	1
Treatment	2	2	0	0
Prognosis	0	0	0	0

Information Classification: General

Table S1. (continued).

	MN patients (n=328)	MN patients with GAg+/SAb+ (n=182)	MN patients with GAg+/SAb- (n=118)	MN patients with GAg-/SAb- (n=28)
Time to partial remission	15	10	3	2
Time to complete remission	9	6	3	0
Relapse	0	0	0	0
ESRD	2	2	0	0
Follow-up time	0	0	0	0

Information Classification: General

Figure S1. Kaplan-Meier analysis for remission based on glomerular PLA2R expression

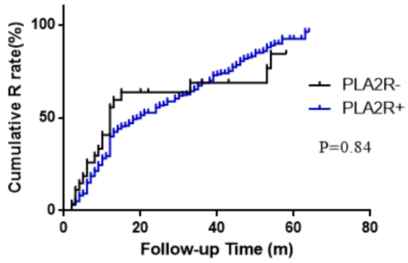

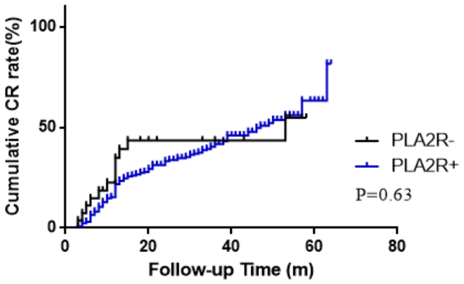

R: remission; CR: complete remission

Information Classification: General
